# Economic Convenience of Hybrid Thermoelectric-Photovoltaic Solar Harvesters

**DOI:** 10.1021/acsaem.1c00394

**Published:** 2021-04-02

**Authors:** Dario Narducci, Bruno Lorenzi

**Affiliations:** Department of Materials Science, University of Milano-Bicocca, Via Cozzi 55, I-20125 Milan, Italy

**Keywords:** hybrid solar harvesting, thermoelectricity, photovoltaics, economic sustainability, renewable energy

## Abstract

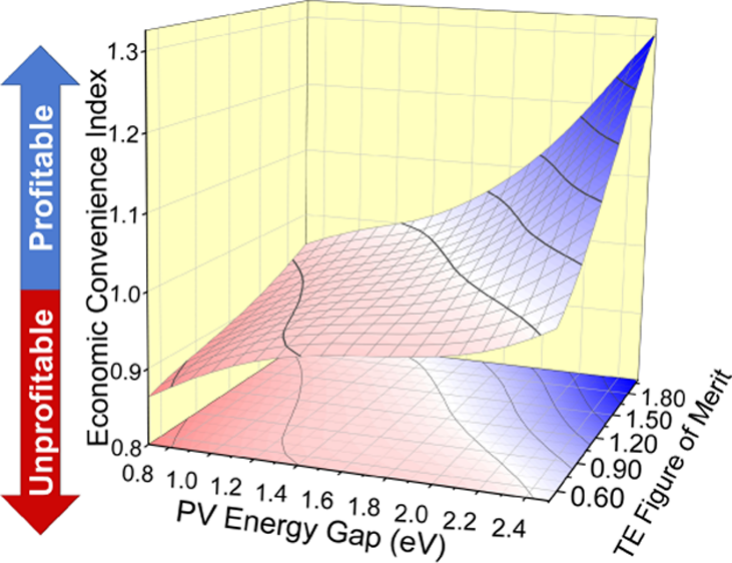

Over the last few years, a growing interest has surfaced about the possibility of enhancing solar harvester efficiency by coupling photovoltaic (PV) cells with thermoelectric generators (TEGs). To be effective solutions, hybrid thermoelectric-photovoltaic (HTEPV) solar harvesters must not only increase the solar conversion efficiency but should also be economically competitive. The aim of this paper is to estimate the profitability of HTEPV solar harvesters with no reference to specific materials, relating it instead to their physical properties only and thus providing a tool to address research effort toward classes of HTEPV systems able to compete with current PV technologies. An economic convenience index is defined and used to assess the economic sustainability of hybridization. It is found that, although hybridization often leads to enhanced solar power conversion, power costs (USD/W) may not always justify HTEPV deployment at the current stage of technology. An analysis of the cost structure shows that profitability requires largely enhanced thermoelectric stages, concentrated solar cells, or PV materials with favorable temperature efficiency coefficients, such as perovskite solar cells.

## 1 Introduction

Photovoltaic (PV) cells are devices capable of converting electromagnetic radiation into electricity. The efficiency of a PV cell is intrinsically limited by the physical mechanism of power conversion^1^ since energy may be converted without losses only when photon energy is equal to the PV absorber band gap. Photons with lower energies are transmitted while photons with higher energies generate hot hole–electron pairs that relax by dissipating the heat energy exceeding the energy gap. Further reduction of efficiency results from technological factors, including hole-pair recombination at defects. Larger conversion efficiencies may be obtained by multiple-junction or tandem cells, pairing more absorbing materials. However, this unavoidably leads to higher fabrication costs and complexity.

Hybrid thermoelectric-photovoltaic (HTEPV) cells are a possible way to enhance solar harvesting efficiency by (partially) recovering the heat dissipated by the PV stage(s) through a thermoelectric (TE) stage. Over the last years, several approaches have been pursued to effectively pair PV and TE stages. Laboratory prototypes of HTEPV generators have been fabricated and tested as well,^2−11^ although hybrid solar harvesters have not yet moved to manufacturing.

The goal of this paper is to provide a comparative estimate of power costs for PV and hybrid solar harvesters built using contact TE-PV pairing with no reference to specific classes of TE and PV materials. An economic convenience index (EcCI) will be proposed to evaluate both concentrated and nonconcentrated HTEPVs. Analysis of power costs will be complemented by an evaluation of the payback period (PBP) of hybrid solar harvesters. This work will focus on single-junction solar cells working at small solar concentrations, which are the dominating technology in the domestic (rooftop) market. Since the analysis that will be proposed relies on the cost structure of the hybrid solar harvesters, multiple-junction solar cells (operating at much larger optical concentrations and mostly aimed at centralized power plants) will not be considered to keep the economic analysis manageable and compendious. Additionally, with multiple-junction solar cells with PV efficiencies commonly exceeding 35%, making HTEPV harvesters profitable would face the challenge of achieving efficiency improvements of several percentage points to pay back the additional costs related to the use of segmented thermoelectric generators (TEGs) operating over extended temperature ranges.

**Figure 1:**
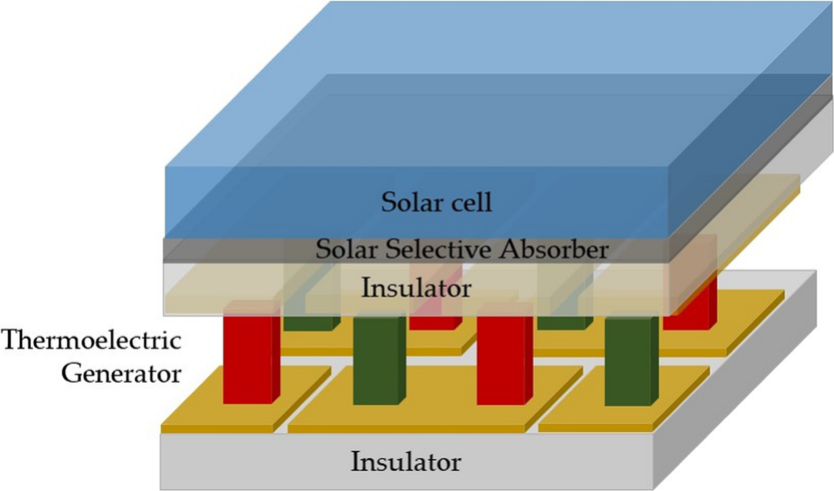
Schematics of a hybrid TE-PV solar harvester implementing direct (contact) pairing between the two stages.

Seemingly, direct (contact) pairing (Figure 1) might not necessarily be the most appropriate approach to hybridization as the TE stage requires high temperatures, while the PV efficiency decays with temperature. Alternate layouts have been considered, including spectrum-splitters^12−14^ and optical coupling.^15^ However, while such pairing schemes avoid temperature compromises between PV and TE stages, they inevitably make unavailable to the TE conversion the large amount of heat released by the PV stage. Furthermore, HTEPV harvesters are not the only possibility to use sun power more extensively.

Beyond energetic convenience, for HTEPV harvesters to be a viable technology, their economic profitability also needs to be assessed. Possibly, the first attempt to estimate HTEPV convenience was advanced by van der Sark.^16^ van der Sark evaluated the affordability of pairing TEGs to PV cells by considering the extra cost of a TEG retrofit sized in such a way to convert all heat released by the PV stage, reaching the conclusion that HTEPV could have attained economic convenience only when TEG costs would have dropped down by at least a factor of 10. More recently, Zhu et al.^17^ computed the power costs χ_HTEPV_ (USD/W) of hybrid harvesters by optimizing costs ahead of power conversion. Economic convenience was properly assumed to occur when the power cost of the HTEPV was lower than that of the PV module, under the additional requirement that the total power output also exceeded a minimum output power set by user-application needs. Unfortunately, Zhu et al. fully neglected the areal cost of installation. In addition, PV efficiency was taken as a constant, not depending on (decreasing with) hybridization. This makes their results valid only for (ideal) spectrum-split solutions.

In more recent years, several papers have been published reporting accurate evaluations of the economic viability of TE hybridization of given classes of PV modules. Integration of the concentrated PV system based on triple-junction cells (GaInP/GaInAs/Ge) with TEGs was analyzed by Rezania and Rosendahl.^18^ Direct pairing was investigated, showing that, for current TE technologies (ZT ≈ 1), further to an improvement of efficiency, suitable choice of heat exchangers makes HTEPV also economically viable. The weight of heat exchanger costs was further considered by Rodrigo et al.,^19^ still investigating highly concentrated solar harvesters and triple-junction solar cells. While high-efficiency, yet unavailable, TEGs disclose excellent opportunities of enhanced conversion, both energetically and economically, a trade-off was searched between cost and efficiency by optimizing the concentration factor, the heat sink thermal resistance, and the TEG area. A predicted cost reduction of about 38% is found at 1900 suns for realistic TEG figures of merit. Unfavorable conclusions were reached instead for nonconcentrated Si-based PV cells^20^ where, compared to stand-alone PVs, an optimal levelized cost of energy higher by 8.7–90% was computed.

A different approach to profitability evaluation was suggested by the present authors,^21^ accounting for the concurrent cost increase due to the TEG stage and the decay of PV performance due to the heating of the PV stage based upon the physical characteristics of PV and TE materials only. In this paper, we will fully develop such a methodology. Its main merit is that it makes economic analyses an additional tool to be used to scout suitable PV-TE material pairs for HTEPV generators. Although the design of efficient HTEPV generators critically depends on a number of factors,^22^ active materials obviously play a pivotal role. We anticipate that the PV material governs the power effectiveness of any hybridization. As mentioned, in most hybridization schemes, the PV cell temperature raises, so the pairing of a PV stage with a TE stage implies either compromises or innovative layouts to be found. While the PV efficiency is commonly large at lower temperatures, TEGs require high temperatures at their hot side and large temperature differences across their legs. This notwithstanding, inorganic PV materials may disclose realistic opportunities of successful hybridization leading to increased total efficiencies. Specifically, performances of large-gap PV materials are less sensitive to the increased temperatures needed by pairing with TEGs. Therefore, concentrated solar cells based on a-Si, Cu_2_ZnSnS_4_ (CZTS), copper gallium selenide CuGaSe_2_, and GaInP may be sensibly expected to be suitable for hybridization, along with stabilized perovskite solar cells. Instead, this rules out some common PV systems (e.g., polycrystalline silicon). Furthermore, low-cost, nontoxic materials with acceptable ZTs at moderate-to-low temperatures are needed for the TE legs.

Materials issues are however not limited to active parts of the two solar stages. As an example, suppressing thermal cross-talk among TE legs is of paramount relevance in solar thermoelectric generators (STEGs),^23^ and very low emittances from leg lateral surfaces also remain important in HTEPV generators. On the PV side, effective heat mirrors limiting the upward radiative dissipation of heat were shown to remarkably enhance the overall efficiency of TEGs.^24^ Furthermore, heat dissipation at the TEG cold side, playing a key role in any TEG, is a formidable challenge still nowadays, calling for innovative materials and advanced surface finishing.^25,26^ However, differently from PV and TE materials, such materials issues do not set conflicting requirements when designing HTEPV cells.

## 2 Cost Structure

Economic convenience of a renewable power technology is set, in general terms, by the capital cost of the generator, namely, the cost of the harvester (including the electronics needed to make the electric power output useable) and of its installation. Therefore, power cost (USD/W), defined as the electric power generated divided by the capital cost of the plant, is the basis of comparison among competing renewable energy technologies. Economic convenience of a power technology integrating an existing renewable power source should be evaluated both in comparison to existing renewable technologies and with respect to all power sources, including nonrenewable ones.

In what follows, the analysis of capital costs of HTEPV generators will be split into installation costs and costs of the HTEPV components.

Power cost is however only a part of the analysis. PBP measures the profitability of a renewable power technology compared to the overall market of power sources (including nonrenewable). PBP is defined as the period of time needed for the energy obtained by the power plant to compensate the plant capital costs. Therefore, PBP depends upon the current energy price. For a technology to be profitable, PBP must be shorter than the average lifetime of the power plant. Therefore, PBP will also be evaluated.

### 2.1 Cost Structure of TEGs

The analysis of the economic sustainability of TEGs has been the subject of several papers.^27−30^ Among them, the approach developed by Yee et al.^30^ is especially suitable for the forthcoming analysis of the HTEPV economic convenience. As observed, optimization of a TEG on its efficiency followed by the search for the lowest possible system cost forces the analysis to pursue the smallest possible cost at the highest peak power while neglecting scenarios where lower peak powers might lead to lower power costs. Therefore, the overnight capital costs were evaluated. Here and in the rest of this paper, we will use double and triple primed symbols to refer to areal and volume costs, respectively. Volumetric module costs *C*_TEG_^‴^ (USD/m^3^), i.e., costs of TEG components scaling with the module volume (e.g., the TE material), were combined with areal module costs *C*_TEG_^″^ (USD/m^2^), i.e., costs of TEG components scaling with the module contact area (e.g., the alumina insulators), and with heat exchanger costs *c*_HX_ (USD/(W K^–1^)). Total TEG cost *C*_TEG_ accounted to
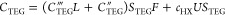
1where *F* is the filling factor, defined as the ratio between the total TE leg area and the TEG footprint area *S*_TEG_, *L* is the leg length, and *U* is the heat transfer coefficient. The power output *P*_TEG_ reads^30^
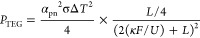
2where κ and σ are the thermal and electric conductivities of the leg elements (assumed to be the same for the p and n elements), α_pn_ ≡ α_p_ – α_n_ (with α_p_ and α_n_ being the Seebeck coefficients of the p and n legs), and Δ*T* is the temperature difference between the two heat reservoirs. Therefore, eqs 1 and 2 immediately return the cost per unit power
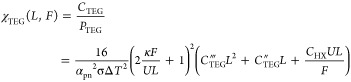
3

Yee et al. showed that eq 3 admits no global minimum on *F* and *UL*/κ. Nonetheless, the χ_TEG_(*L*, *F*) surface displays a narrow region around the line *F* = *UL*/(2κ) where χ_TEG_ takes low values, resulting from a competition between costs and TE performances.^30^ For smaller *L* (at constant *F*), costs decrease along with the temperature drop across the device, so that power output also decreases. Furthermore, a characteristic point exists
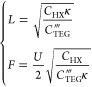
4below which any further decrease of *L* and *F* has only marginal benefits on χ_TEG_. Exemplar values for *C*_TEG_^″^, *C*_TEG_^‴^, and *C*_HX_ lead to optimal χ_TEG_ around 60 USD/W, including the large contribution arising from the heat exchanger.

Of special relevance to hybrid solar generators is the scenario wherein no heat exchanger cost adds up. Thus eq 3 simplifies to
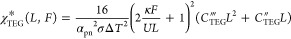
5Manifestly enough, neither a characteristic point nor a line of minimal cost exists any longer for *C*_HX_ = 0 in eq 4. It is instead remarkable that lower χ*_TEG_ may be achieved for leg lengths *L* shorter than the thermal-impedance-matching value 2*F*κ/*U*, showing how more favorable power costs may be obtained for TEGs operating under nonoptimized conditions. Economic convenience stems from lower material costs overcompensating the reduced power output.

### 2.2 Cost Structure of PV Harvesters

Cost structure of PV plants, a current deployed technology, is paradoxically more complex to analyze than that of TEGs, namely a forthcoming technology. This is basically due to the rapid changes of the cost structure, redistributing costs across diverse technology elements. We will focus this analysis on rooftop (domestic) solar plants, with typical power outputs below 20 kW. In this class of harvesters, PV capital costs may be split into four main components, namely, PV materials, substrates (commonly glass), labor, and OEM costs. The dominating technology based on polycrystalline silicon reports rooftop module costs of 0.85 USD/W, to which about 1.00 USD/W must be added due to the balance-of-system (BoS) costs, accounting to 0.12 USD/W for the inverter, 0.08 USD/W for wiring and transformers, 0.05 USD/W for electrical installation, 0.002 USD/m^2^ for site preparation (strongly dependent on the geographical location), mounting, and structural installation, with the complement to the BoS due to business costs.^31^ Business costs will be disregarded in what follows, since we focus on overnight capital costs only. Total costs and cost structures quite differ for other PV materials, although module costs quite line up to polycrystalline silicon modules. For CuIn_*x*_Ga_1–*x*_Se_2_ (CIGS), module costs are reported to range from 0.63 to 0.69 USD/W, while CdTe modules are quoted at 0.49 USD/W.^31,32^ Slightly lower costs imply lower efficiencies (10–13% for CIGS, 16–17% for CdTe compared to 19–20% for polycrystalline silicon) that require larger module areas per output watt. In the forthcoming analysis, we will split BoS costs as costs per watt χ_BoS,1_ (including the inverter, wiring, transformers, and electrical installation) and areal costs *C*_BoS,2_^″^ (encompassing site preparation, mounting, and structural installation).

### 2.3 Projected Cost Structure of Hybrid Solar Harvesters

Based on the framework set up for TEG and PV modules, economic convenience of hybrid solar harvesters may be evaluated.

A full economic analysis of HTEPV generators is a very complex task for several reasons. First of all, layouts to pair PV and TE stages are extremely diversified, encompassing direct thermal contact, thermal concentration, and solar-splitting strategies. In addition, power output from HTEPV critically depends on many subtle constructive details.^22,33^ This was clearly proved for STEGs, where improvements of power outputs by a factor of 3 were achieved by accurately minimizing thermal shunts in the generator.^34^ Therefore, any economic estimation based upon computational models might easily turn out to be overoptimistic, especially in view of the low technological maturity of HTEPV technologies. Furthermore, TEG market is still modest and mostly limited to Bi_2_Te_3_-based systems, so that cost estimates might turn out to be prospectively inaccurate. This said, hybrid harvesters must meet two requirements to be profitable. On the one side, their power costs must be lower than the power cost of their associated PV technology, so that hybridization leads to an economic advantage for the final user. Furthermore, and independently, power costs must also be competitive with those of leading renewable technologies, currently set forth by polycrystalline silicon (pcSi) PV plants.^35^

In what follows, we will assume that the HTEPV structure may be summarized as made of a PV cell in contact with a TEG—either directly or through a layer, referred to as the solar selective absorber (SSA), that acts as a black body converting all electromagnetic radiation transmitted by the PV cell. We further assume that the whole device be suitably encapsulated, so as to prevent convective heat dissipation. Furthermore, radiative dissipation from the PV cell is fully blocked by a suitable heat mirror, namely, a coating on the part of the encapsulating package facing the sun that reflects electromagnetic radiation emitted by the PV cell (mostly in the infrared range) while transmitting solar radiation.^36^ This is a key point that must be stressed. Often, radiative dissipation is disregarded in the thermal budget analysis. In the absence of heat mirrors, instead, a major amount of heat is irradiated, even at relatively low temperatures (cf. Supporting Information and ref (37)). Therefore, effective heat mirrors are not needed to dissipate a significant fraction of heat that may be converted by the TEG stage. The harvester is completed by a heat exchanger, dissipating the heat rejected by the TEG.

To compute the projected power costs of HTEPV harvesters, we will move from the following assumptions.

1. The costs of the BoS, *C*_BOS_, will be split in two parts: one, roughly proportional to the power output, will account for the electronics (inverter, wiring, transformers, and electrical installation), while costs encompassing site preparation, mounting, and installation will be set proportional to the module footprint. Thus

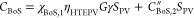
6where *G* is the solar input power density, γ is the optical concentration, and η_HTEPV_ is the efficiency of the HTEPV harvester;

2. The cost of the PV cell will scale with the nonhybridized PV power output, that is, with its area *S*_PV_

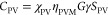
7where χ_PV_ is the PV cost per watt and η_PVM_ is the efficiency of the PV module;

3. The cost of the TE stage will be computed in view of its areal and volume costs through eq 1;
4. Additional costs will arise from the SSA, scaling with its area *S*_SSA_

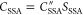
8

5. Costs for the heat exchanger will depend on the heat flux to be dissipated through its heat transfer coefficient *U* and the technology it is based upon, namely

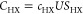
9where *S*_HX_ is its area and *c*_HX_ units are USD/(W/K).

Therefore, assuming hereafter *S*_PV_ = *S*_TEG_ = *S*_SSA_ = *S*_HX_ ≡ *S*
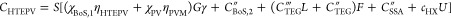
10that compares to the cost of the nonhybridized PV module
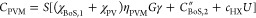
11Cost parameters used as a reference in this paper are displayed in Table 1 and refer to silicon PV cells and Bi_2_Te_3_ TEGs. The effect of their variations for alternate materials will be analyzed in Section 5.3. The choice of polycrystalline silicon PV modules as a cost reference also lets fulfill the second and more general requirement that HTEPV cells must be economically viable when compared to the standard solar technology, which is dominated by (nonhybridized) polycrystalline silicon.

**Table 1:** Cost Parameters Used in the Evaluation of Economic Convenience^a^

parameter	value	parameter	value
*C*‴_TEG_	0.89 USD/cm^3^	*C*_TEG_^″^	0.017 USD/cm^2^
*c*_HX_	10.00 USD/(W/K)	*C*_SSA_^″^	0.001 USD/cm^2^
χ_BoS,1_	0.25 USD/W	χ_PV_	0.85 USD/W
*C*_BoS,2_^″^	0.002 USD/m^2^		

a Data from refs.^17,30,31,35,38^

## 3 Efficiency and Power Output Computation

In this section, efficiency and power output for hybridized (HTEPV) and nonhybridized (PV) solar harvesters will be computed. We would like to stress once again that the present effort to compute power outputs for a yet-to-be technology is unavoidably frustrated by many factors ruling the actual efficiency of hybrid solar harvesters. Thus, the computations we propose are to be meant as a best-case evaluation for domestic (rooftop) solar harvesters. Therefore, they should be used as a no-go criterion, namely, showing when pairing between PV and TE stages may not be profitable, even in the absence of any factor further degrading the efficiency gain.

### 3.1 HTEPV Harvesters

To estimate the power output of the HTEPV harvester, it is convenient to rewrite the TEG power output eq 2 by correlating Δ*T* with the heat flux through the TEG. Neglecting any lateral heat dissipation (due to the encapsulation) as well as the (small) fraction of heat converted into electric power by the TEG,^39^ the temperature drop across the TEG accounts to ϕ′/*K*_TEG_, where ϕ′ is the heat flux input from the PV stage into the TEG and *K*_TEG_ is the thermal conductance of the TEG. It simply computes to
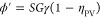
12It may be worth to stress that the previous equation deliberately neglects many possible sources of heat dissipation, including TEG lateral dissipation (due to the encapsulation) and the heat re-emitted by the PV upward^22^ (namely, assuming unitary heat mirror efficiency^37^). Therefore, ϕ′ is a best-case estimation of the real heat flux converted by the TEG, in accordance with the general purpose of this work.

For a TEG made of *N* legs of length *L* and cross-section *A*, if κ is the thermal conductivity of the legs (assumed equal for the p and n legs), then
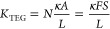
13where we used the relation *F* = *NA*/*S*. The power output of a TEG is *P*_TEG_ = α_pn_^2^Δ*T*^2^/(4*R*_TEG_) where the electrical resistance *R*_TEG_ reads 4*L*/(σ*SF*), with the factor 4 accounting for the series electrical connection of the legs.^30^ Thus
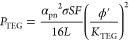
14In view of eqs 12 and 13, the power output may be rewritten as
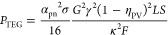
15Note that the formula differs from that obtained by Yee et al.^30^ since here the computation is carried out by setting the temperature of the hot side along with the heat flux at the cold side. It is also remarkable that the power output is formally independent of the thermal resistance of the heat exchanger, which enters instead to set the temperatures *T*_1_ (and *T*_2_) of the hot and cold sides of the TEG.

The efficiency of the single-junction PV stage along with its dependence on γ, *E*_g_, and its technological readiness is computed according to standard models (cf. Supporting Information for details). It accounts to
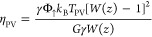
16where *z* ≡ *e*γEREΦ_↑_/*r*_0_, ERE is the external radiative efficiency,^40^*e* is the Neper’s number, *r*_0_ is the radiative recombination eq S1, Φ_↑_ is the flux of photons with *E* > *E*_g_ at 1 sun eq S6, *k*_B_ is the Boltzmann constant, and *W*(*z*) is the Lambert function. Note that η_PV_ depends on *T*_PV_. Replacing it into eq 12, the temperature difference *T*_PV_ – *T*_a_ (where *T*_a_ is ambient temperature) may be obtained by using Fourier equation under the sensible assumption that the temperature of the PV cell is equal to that of the hot side of the TE stage, namely, *T*_PV_ = *T*_1_. Since
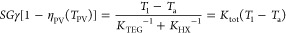
17(with *K*_tot_^–1^ = *K*_TEG_^–1^ + *K*_HX_^–1^), replacing *K*_HX_ with *US*, one obtains
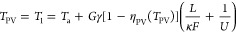
18that may be used to compute *T*_PV_.

Assuming that each generator provides its output to an independent, optimized electric load, the total power output reads *P*_HTEPV_ = *P*_PV_ + *P*_TEG_ = η_HTEPV_*SG*γ and leads to
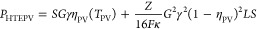
19so that
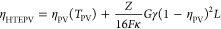
20

### 3.2 Nonhybridized Photovoltaic Harvesters

For the standard PV module, computations simplify. Fourier equation reads in this case
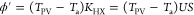
21Thus, the power output is
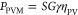
22while the efficiency is the same as reported in eq 16, namely,
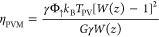
23although it must be emphasized that in this case, *T*_PV_ is lower than in the hybrid device due to the lack of the TEG and its contribution to the total thermal resistance.

## 4 EcCI

In a previous paper,^37^ one of the present authors introduced an energetic convenience index to evaluate the energetic advantage of pairing PV and TEG generators into a hybrid solar harvester. In what follows, an EcCI will be defined and analyzed to establish the economic profitability of such a coupling.

**Figure 2:**
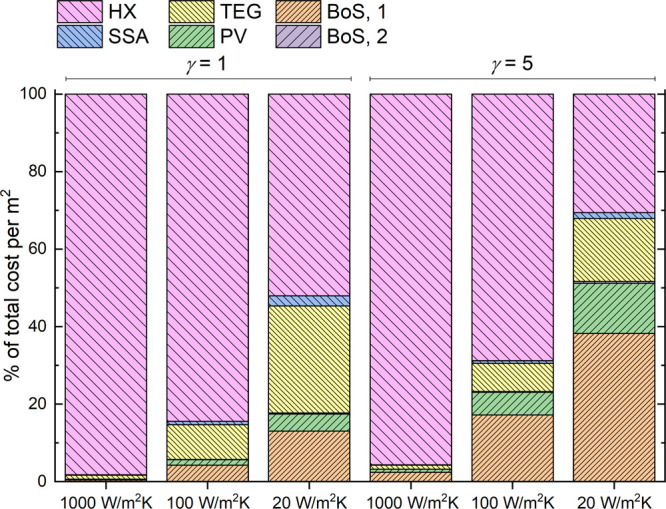
Details of the cost structure of HTEPV harvesters for nonconcentrated (γ = 1) and concentrated (γ = 5) solar converters equipped with exemplary heat sinks. Heat transfer coefficients range from *U* = 20 W/m^2^K (air cooling) to *U* = 1000 W/m^2^K (high-efficiency water cooling).

Before proceeding to the analysis of the EcCI, some preliminary comments may be in order. Figure 2 shows how various components enter into setting the HTEPV cost for an exemplary system. Based on eq 10 and for *F* = 0.1, *L* = 0.1 cm, and η_HTEPV_ = η_PVM_ = 0.2, one may note how the dominating cost factors are in all cases those of components which are not directly related to the harvesters, since BoS and the heat exchanger mostly set the power costs.

The power cost (USD/W) of the HTEPV harvester reads eq 10
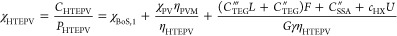
24where we neglected the truly marginal contribution coming from *C*_BoS,2_^″^.

The corresponding power cost for the nonhybridized PV module is instead eq 11
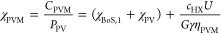
25

An EcCI is defined as
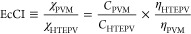
26Hybridization is profitable for EcCI >1.

## 5 Results and Discussion

### 5.1 HTEPV Optimization and Technological Constraints

The EcCI is a multivariable function depending on material parameters (*Z*, *E*_g_, and the ERE), on constructive specifications (*L*, *F*, and *U*), and on the operative conditions (γ). Optimization (maximization) of EcCI on the constructive parameters leads therefore to maximal profitability. It is easy to verify that χ_HTEPV_ (eq 24) is a decreasing, unbound function of *L*/(κ*F*). In most cases, however, it is technology to set limits to both *L* and *F* values since leg lengths in excess of 1 cm are possibly too critical to manufacture and use, and filling factors larger than 0.1 would induce large radiative interleg cross-talking.^30^ Furthermore, EcCI is found to admit a maximum for large *U* values. However, such values largely exceed any realistic capability of heat dissipation by conventional exchangers. Thus, a limit of 10^4^ W/m^2^K has been stipulated in all computations. As a result, optimized EcCI ultimately depends on the materials parameters and on solar concentration only. Consequently, we split optimal EcCI analysis into two major cases, namely, concentrated and nonconcentrated harvesters.

### 5.2 EcCI at Standing Technology and Costs

**Figure 3:**
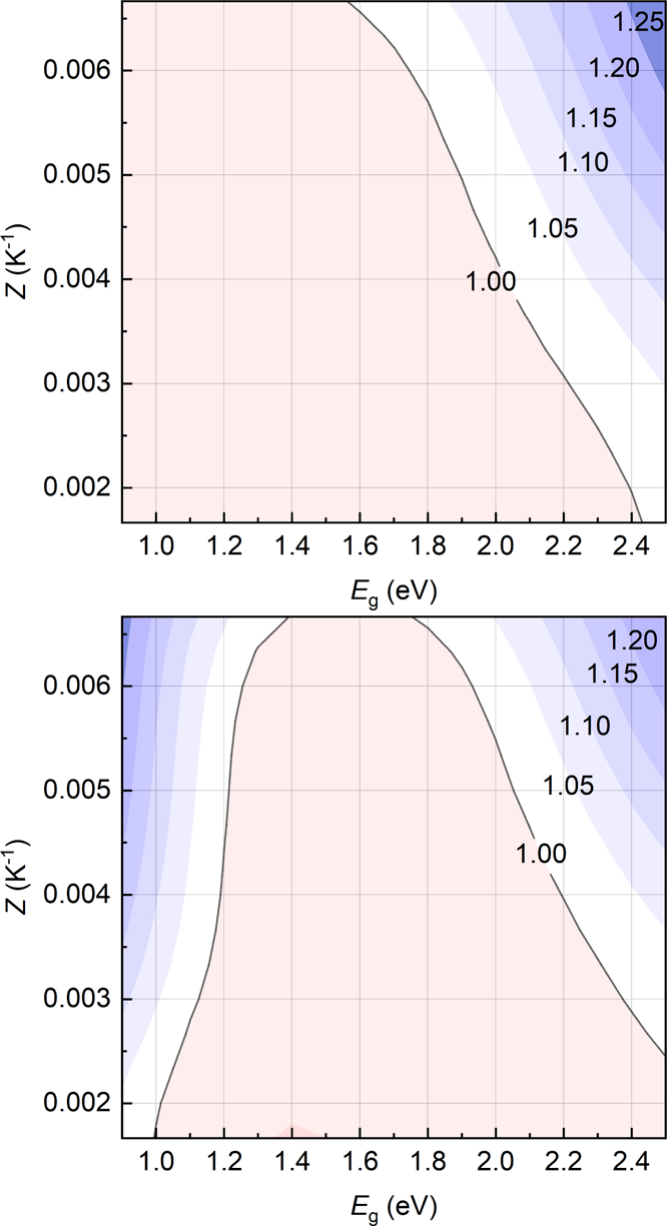
Contour plots of the optimized EcCI of nonconcentrated hybrid solar harvesters for ERE = 1 × 10^–2^ (top) and 1 × 10^–6^ (bottom) as a function of the PV energy gap and of *Z*. Cost parameters are as reported in Table 1.

Inspection of Figure 3 shows that for nonconcentrated (γ = 1) HTEPV generators, a window of convenience shows up for wide-gap PV materials and top-performing, state-of-the-art TE materials (with *Z* = 0.004 K^–1^). Beyond the real availability of TEGs with such high performances, in all cases, the increase of economic profitability is truly marginal. For realistic *Z* values of ≈0.003 K^–1^, EcCI is larger than unity only for *E*_g_ > 2.2 eV in technologically mature PV materials (ERE = 1 × 10^–2^). For less optimized PV absorbers, instead, EcCI exceeds 1 only for *E*_g_ > 2.4 eV. This is sensible since for low EREs, the heating of the PV stage is more severe, and larger energy gaps are needed to make the reduction of PV efficiency less severe. The dominating role of the PV stage at low EREs and current *Z* values is confirmed considering that, instead, at (today unrealistically) large *Z*s, EcCI >1 for almost any *E*_g_ value since extremely efficient TEGs would enable TE power generation overcompensating the decrease of PV efficiency.

For concentrated solar harvesters, analyses will be limited to the case of γ = 5, a typical concentration rate for rooftop solar plants.

**Figure 4:**
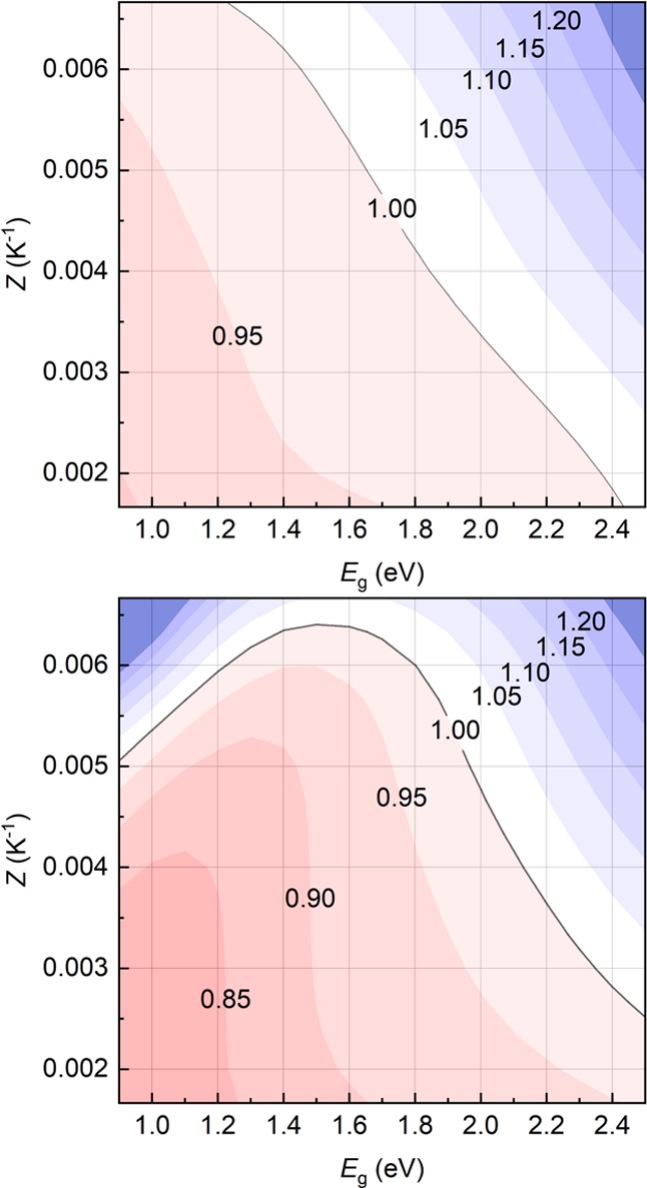
Contour plots of the optimized EcCI of 5-sun-concentrated hybrid solar harvesters for ERE = 1 × 10^–2^ (top) and 1 × 10^–6^ (bottom) as a function of the PV energy gap and of *Z*. Cost parameters are as reported in Table 1.

In concentrated generators, EcCI shows slightly larger margins (Figure 4). For currently realistic TE efficiencies, EcCI > 1 for fully engineered PV materials (ERE = 1 × 10^–2^) requires *E*_g_ > 2.1 eV. As in the nonconcentrated case, less-performing PV absorbers (ERE = 1 × 10^–6^) need instead larger energy gaps (>2.3 eV) to attain EcCI > 1.

For polycrystalline silicon, a key player in the PV technology, it follows that in all cases, one computes EcCI < 1 for *Z* = 0.003 K^–1^. Rather, a wide-gap PV material such as CdS (*E*_g_ = 2.4 eV) would benefit from PV-TE pairing, with EcCIs ranging from 1.003 (low ERE, γ = 1) to 1.01 (low ERE, γ = 5), reaching 1.026 and 1.050 for high ERE and PVs at γ = 1 and 5, respectively. It should be stressed that an EcCI of 1.05, although seemingly meager, is instead equivalent to an efficiency enhancement of 5% of solar conversion efficiency at a constant price. Thus, although profit margins are limited, one may conclude that rooftop concentrated solar harvesters might be anyway a possible driver to support the initial development of the HTEPV technology using novel, low-cost wide-gap PV materials—with an advantage when moderate solar concentration is considered. This is especially interesting if one considers that rooftop panels are among the most rapidly expanding market niches in the sector of renewable energies and that profitability may be eventually enhanced by triple cogeneration, with sanitary hot water being made available in addition to electric power production.^41^

### 5.3 EcCI Enhancement following Cost Rebates

It is interesting to consider how a prospective cost rebate of single parts of the technologies involved in HTEPV would impact the EcCI.

Numerical estimates show that reductions up to 50% of volumetric module costs (*C*_TEG_^‴^) would lead to an absolute EcCI improvement of ≈0.004. Even smaller is the impact of a similar reduction of areal costs (*C*_TEG_^″^), causing EcCI to increase by ≈0.001. In both cases, changes of EcCI are comparable independently of the PV energy gap, ERE, and solar concentration. It is instead possibly unexpected that savings up to 50% of exchanger costs per W/K (*c*_HX_) would worsen EcCI—by about 0.04 for γ = 1 and by about 0.005 for γ = 5. This is basically due to the fact that savings are more relevant to PV modules. Thus, even if HTEPV costs would obviously decrease, nonetheless they would decrease more in nonhybrid PV modules, and therefore EcCI would worsen. Stated differently, a rebate of the exchanger cost would improve EcCI only if the TE module had a significantly larger efficiency.

### 5.4 EcCI for Perovskite-Based Solar Cells

Perovskite-based solar cells (PSCs) display a remarkably different dependence of their efficiency upon temperature and solar concentration as the underlying physics of solar conversion in PSC somewhat differs from ordinary solar cells.^42^ Despite well-known, yet partially solved issues with their stability,^43,44^ PSCs are nonetheless taking a major role in solar conversion and their possible pairing with TEGs is therefore worth to be considered.

**Figure 5:**
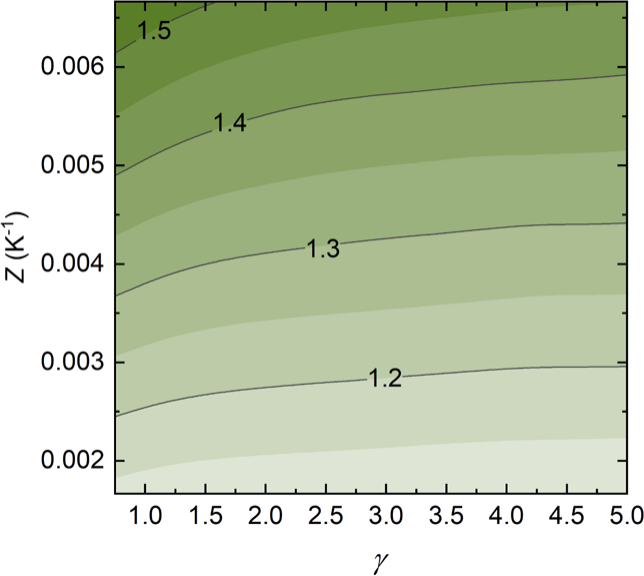
Contour plots of the optimized EcCI of an exemplar perovskite hybrid solar cell as a function of the solar concentration γ and of *Z*.

Recent experimental analyses^45,46^ have shown that the PSC efficiency, instead of decreasing, increases with temperature, reaching a maximum in the temperature range 45–55 °C, then rapidly dropping at higher temperatures. This is consistent with a tetragonal-to-cubic phase transition reported for methylammonium lead iodide perovskites.^47−49^ Furthermore, the efficiency was shown to increase with optical concentration (from 1 to 5 suns).^50^ Thus, EcCI was computed using data for a specific PSC^46,50^ adding the constraint that *T*_PV_ < 323 K. Figure 5 shows that in this case, EcCI is remarkably larger than unity at any solar concentration even at currently reachable *Z* values. Since estimates of cost parameters for PSCs are largely variable, we inherited the same cost parameters used for the whole analysis, namely, those typical of silicon solar cells (Table 1), to validate its convenience also compared to current PV polysilicon technologies. Nonetheless, rebates or cost increases up to ±50% do not change the conclusions, showing that perovskites have a great potential for TE hybridization, as anticipated by several scholars.^45,51−53^

### 5.5 PayBack Period

For the sake of completeness, it should be mentioned that an additional index of affordability for PV cells is the PBP, namely, the period of time needed to pay back the capital cost. PBP should not be confused with the energy payback period, which counts instead the period of time needed to generate the amount of energy spent to build the PV system.^54^ While PBPs are currently cut down by taxation and other supporting benefits, true PBPs are easily computed to be around 20 years for both domestic and industrial solar power plants.^55^ In hybrid solar harvesters, PBPs scale with EcCI and with the ratio of HTEPV to PV capital costs. In the case of concentrated solar cells, this leads to an increase of PBP of less than 5% in the worst case. Since TEGs are known to have a very extended lifetime, much larger than that of the solar cells, hybridization retains acceptable PBPs even in the absence of favorable taxation.

### 5.6 Conclusive Remarks

The results reported in the present analysis outline the windows of expediency opened by research on hybrid solar harvesters. Specifically, the criteria advanced in the previous section enable to address technology exploitations toward a well-defined applicative context and market. While for nonconcentrated solar harvesters, TE efficiencies are too small to enable significant margins of profit, hybridization applied to civilian concentrated solar converters is found to be viable and convenient.

This conclusion is relevant both to PVs and to TEs. From the viewpoint of novel PV materials, hybridization may shorten their time-to-market, compensating for the relatively low EREs. Concerning TEs, instead, their deployment in conjunction with well-established and socially accepted PV technologies might support the overall technology readiness of TEGs, promoting the development of new materials capable of acceptable efficiencies at intermediate temperatures.^15,56^

A final point is possibly worth to be stressed again. When applied to blue-sky research, EcCI should always be used as a no-go criterion. Equation 26, although accounting for materials quality through the ERE, fully neglects thermal shunts and other dissipation mechanisms in the TEG stage and additional PV losses such as those due to radiation scattering and re-emission in the PV stage.^37^ More accurate estimations are instead possible for existing technologies, where EcCI is computed directly through eqs 24 and 25.

## 6 Summary and Conclusions

A scheme to evaluate the profitability of harvesters based upon generic PV and TE materials operated at small solar concentrations has been developed.

The comparative analysis of the current and perspective possibilities provided by the hybridization of TE and single-junction PV generators has shown the existence of some windows of economic convenience. Specifically, it has been shown how at current PV costs and for realistic TE efficiencies, hybridization might be viable for rooftop concentrated solar harvesters only. Far more promising scenarios are envisaged with PSC-TE hybrid cells.

The overall landscape we outlined should address research along two directions. Further to the prospective that would be opened by the availability of TEG modules with higher efficiencies, HTEPV could enable the concurrent use of TEs and low-efficiency, lower-cost PV materials, promoting at one time the diversification of PV materials and reopening application interests toward age-old materials such as *a*-Si or Cu_2_O. Hybridization would support their technological development (increase of ERE) by anticipating their commercial viability to a widely expanding market as that of domestic solar harvesting.

On the other side, perovskites—and possibly other new PV materials showing a temperature range wherein the PV efficiency remains constant or even increases with temperature—open great prospects for a beneficial and cost-effective TE hybridization.

## Supporting Information Available

Validation of models used to compute the PV efficiency (PDF)
