# Supplementary material for: Economic Convenience of Hybrid Thermoelectric-Photovoltaic Solar Harvesters
Source: ACS Appl Energy Mater. 2021 Apr 2;4(4):4029–37. doi: 10.1021/acsaem.1c00394 (PMC8159161; doi:10.1021/acsaem.1c00394)
Supplement: Supplementary file 1 — ae1c00394_si_001.pdf [file ae1c00394_si_001.pdf]

# Economic convenience of hybrid photovoltaic-thermoelectric solar harvesters

# Supporting Information

Dario Narducci* and Bruno Lorenzi,
Department of Materials Science, University of Milano-Bicocca,
via Cozzi 55, I-20125 Milan, Italy

* Corresponding author. E-mail: dario.narducci@unimib.it

Aim of the models we report in what follows is to allow PV efficiency to be related to materials characteristics, namely its energy gap $E_{\mathrm{g}}$ and its ERE; and to its temperature $T_{\mathrm{PV}}$ as well.

## Dependence of the photovoltaic efficiency on $E_{\mathrm{g}}$, $T_{\mathrm{PV}}$, $\gamma$, and the materials technological readiness

Efficiency of an ideal single-junction solar cell follows from the Shockley-Queisser (SQ) model [6], which provides a first account of the dependence of $\eta_{\mathrm{PV}}$ on the materials energy gap and on the cell temperature. The SQ model encompasses only for radiative recombination:

$$r_0(E_{\mathrm{g}}, T_{\mathrm{PV}}) = \frac{2\pi}{c^2 h^3} \int_{E_{\mathrm{g}}}^{\infty} \frac{E^2}{\exp[E/(k_{\mathrm{B}} T_{\mathrm{PV}})] - 1} \mathrm{d}E \tag{S1}$$

where $c$ is the speed of light, $k_{\mathrm{B}}$ is the Boltzmann constant and $h$ is the Planck constant. The radiative recombination rate may be integrated analytically to give

$$r_0(E_{\mathrm{g}}, T_{\mathrm{PV}}) = \frac{2\pi (k_{\mathrm{B}} T_{\mathrm{PV}})^3}{c^2 h^3} \Theta\left(\frac{E}{k_{\mathrm{B}} T_{\mathrm{PV}}}\right) \tag{S2}$$

where

$$\Theta(z) := -z^2 \log(1 - e^{-z}) + 2z\mathrm{Li}_2(e^{-z}) + 2\mathrm{Li}_3(e^{-z}) \tag{S3}$$

and $\mathrm{Li}_n(z)$ is the Jonquière's function of order $n$:

$$\mathrm{Li}_n(z) := \frac{1}{\Gamma(n)} \int_0^{+\infty} \frac{t^{n-1}}{e^t/z - 1} \mathrm{d}t \tag{S4}$$

ang $\Gamma(n)$ is the Gamma function.

In real devices, non-radiative recombination not only scales down $\eta_{\mathrm{PV}}$ but also re-modulate its degradation with cell temperature.

A simple, yet effective way to include non-radiative recombination in the computation of a PV cell efficiency was proposed by Green [3], who introduced the concept of external recombination efficiency (ERE), ranging from $10^{-3}$ – $10^{-4}$ for single-crystalline silicon down to $10^{-6}$ for CIGS (copper indium gallium sulfide). Current density reads then

$$J(V, E_\mathrm{g}, \mathrm{ERE}, T_\mathrm{PV}, \gamma) = q\left(\gamma\Phi_\uparrow(E_\mathrm{g}) - \frac{r_0(E_\mathrm{g}, T_\mathrm{PV})}{\mathrm{ERE}}\exp\left(\frac{qV}{k_\mathrm{B}T_\mathrm{PV}}\right)\right) \tag{S5}$$

where $-q$ is the electron charge and $V$ is the voltage,

$$\Phi_\uparrow(E_\mathrm{g}) = \int_{E_\mathrm{g}}^{\infty} n_\mathrm{ph}(E)\mathrm{d}E \tag{S6}$$

is the flux of photons with $E > E_\mathrm{g}$ and $n_\mathrm{ph}(E)$ is the incident spectral photon flux (in $\mathrm{eV}^{-1}\mathrm{m}^{-2}\mathrm{s}^{-1}$), both at one sun. A standard AM1.5 solar spectrum was used throughout all computations.

Short-circuit current density $J_\mathrm{sc}$ follows by setting $V = 0$ V in Eq. (S5) while the open-circuit voltage $V_\mathrm{oc}$ reads

$$V_\mathrm{oc} = \frac{k_\mathrm{B}T_\mathrm{PV}}{q}\ln\left(\frac{\Phi_\uparrow}{r_0(E_\mathrm{g}, T_\mathrm{PV})} + 1\right) \tag{S7}$$

Maximum power output is obtained for $V$ values maximizing $J \times V$ [4], namely

$$V_\mathrm{m} = \frac{k_\mathrm{B}T_\mathrm{PV}}{q}[W(z) - 1] \tag{S8}$$

where $z := \gamma e \mathrm{ERE}\Phi_\uparrow / r_0(E_\mathrm{g}, T_\mathrm{PV})$, $e$ is the Napier's number (base of the natural logarithm), and $W(z)$ is the Lambert function (product logarithm), defined as the inverse function of $f(W) = We^W$.

Therefore, the power output of a real PV cell may be written as a function of the energy gap of the PV absorber, of the ERE, and of the cell temperature, as:

$$P_\mathrm{PV} = \frac{\gamma\Phi_\uparrow k_\mathrm{B}T_\mathrm{PV}[W(z) - 1]^2}{W(z)} \tag{S9}$$

Thus,

$$\eta_\mathrm{PV} = \frac{\gamma\Phi_\uparrow k_\mathrm{B}T_\mathrm{PV}[W(z) - 1]^2}{G\gamma W(z)} \tag{S10}$$

Despite its simplicity, the model accounts more than fairly for the dependence of the PV efficiency on ERE, $E_\mathrm{g}$, $\gamma$, and $T_\mathrm{PV}$. Figure S1 displays a comparison between the efficiency as a function of the energy gap and of the ERE as computed using literature and current models [3]. The agreement is almost perfect. The figure also displays experimental efficiencies for solar cells based upon various materials, providing a confirmation of the suitability of ERE as a measure of technological materials qualification.

Additional validation of the model could be obtained by analyzing the predicted degradation of the PV power output upon increasing cell temperature in non-concentrated solar cells. Reduction of efficiency is found in good agreement with experimental data [2] (Fig. S2). Solar concentration is reported to mitigate the degradation of efficiency with $T_\mathrm{PV}$ [5]. Also for this second-order yet relevant effect, the model is found to be in fair agreement with experimental data. Figure S3 shows the combined effect of solar concentration (and of the consequent temperature increase) on the PV efficiency.

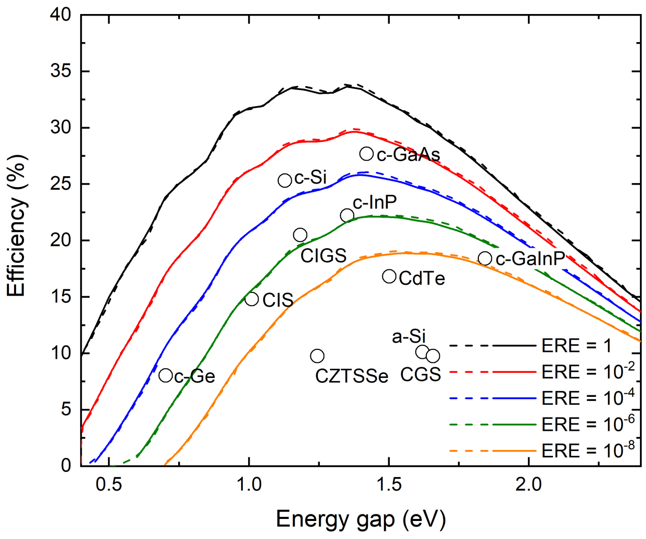


Figure S1: Dependence of the PV efficiency of single-junction solar cells on their energy gap $E_g$ for various ERE values. Markers display experimental efficiencies of specific PV materials while dashed lines reports the efficiency predicted in literature [3]. Full lines show instead the results of the model used in this work.

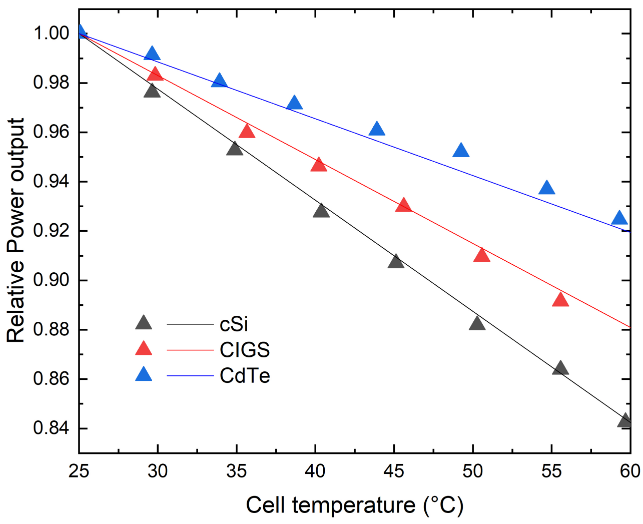


Figure S2: Predicted degradation of the PV power output upon increasing cell temperature. Experimental data from Dupré *et al.* [2].

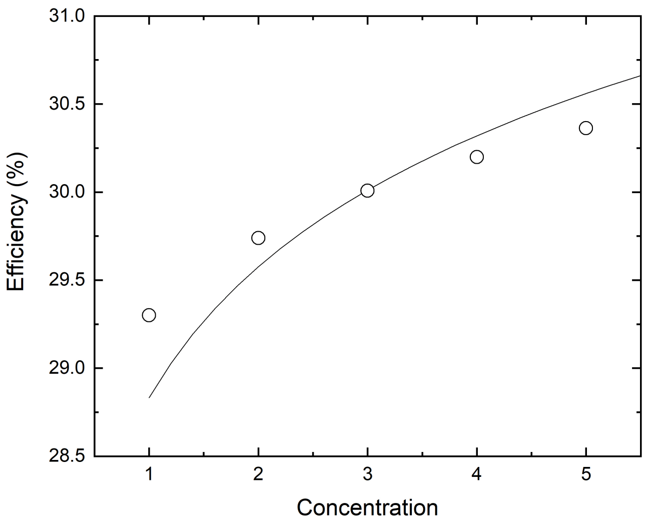


Figure S3: Exemplar variation of the PV efficiency vs. solar concentration. Experimental data from Campbell & Green [1].

# References

[1] P. Campbell and M. A. Green. The limiting efficiency of silicon solar cells under concentrated sunlight. *IEEE Transactions on Electron Devices*, 33(2):234–239, Feb 1986.

[2] O. Dupré, R. Vaillon, and M. A. Green. *Thermal Behavior of Photovoltaic Devices*. Springer International Publishing, 2016.

[3] M. A. Green. Radiative efficiency of state-of-the-art photovoltaic cells. *Progress in Photovoltaics: Research and Applications*, 20(4):472–476, 2012.

[4] B. Lorenzi, M. Acciarri, and D. Narducci. Conditions for beneficial coupling of thermoelectric and photovoltaic devices. *J. Mater. Res.*, 30(17):2663–2669, 2015.

[5] B. Lorenzi and G. Chen. Theoretical efficiency of hybrid solar thermoelectric-photovoltaic generators. *J. Appl. Phys.*, 124(2):024501, 2018.

[6] W. Shockley and H. J. Queisser. Detailed balance limit of efficiency of p-n junction solar cells. *J. Appl. Phys.*, 32(3):510–519, 1961.
